# Clinical results of arthroscopic tight fibrous band release for adult moderate-to-severe gluteal fibrosis using anterior and posterior portals: a retrospective analysis of 118 consecutive cases

**DOI:** 10.1186/s12891-020-03885-z

**Published:** 2021-01-06

**Authors:** Shu-guang Gao, Wei-jie Liu, Ming Yang, Jing-ping Li, Chao Su, Shi-da Kuang, Jie-peng Xiong, Ke Chou, Zhi-yong He, Liang-jun Li

**Affiliations:** 1 Department of Orthopaedics, Xiangya Hospital, Central South University, 87 Xiangya Road, Changsha, 410008 Hunan China; 2 National Clinical Research Center of Geriatric Disorders, Xiangya Hospital, Central South University, Changsha, China; 3 Hunan Key Laboratory of Joint Degeneration and Injury, Changsha, China; 4 Department of Orthopaedics, Changsha Central Hospital, University of South China, 161 Southern Shaoshan Road, Changsha, Hunan China

**Keywords:** Gluteal fibrosis, Gluteal muscle contracture, Arthroscopy, Surgical treatment, Adult

## Abstract

**Background:**

To evaluate the clinical outcomes of arthroscopic tight fibrous band release in the treatment of adult moderate-to-severe gluteal fibrosis using anterior and posterior portals during mid-term follow-up.

**Methods:**

The data of 138 patients (58 males, 80 females) aged between 18 and 42 years (mean, 28.6 years), presenting with bilateral moderate-to-severe gluteal fibrosis (GF) from October 2013 to August 2019, was retrospectively analyzed. All patients underwent arthroscopic tight fibrous band release using anterior and posterior portals with radiofrequency energy. Under arthroscopic guidance through the posterior portal, we debrided the fatty tissue overlying the contracted band of the gluteal muscle and excised the contracted bands using a radiofrequency device introduced through the anterior portal. The pre- and post-operative gluteal muscle contracture disability (GD) scale and the patient satisfaction rate were compared to evaluate the curative effect of the operation.

**Results:**

The average operation time was 18 min (range, 10–30 min) and the average blood loss was 4 ml (range, 2–10 ml) for unilateral arthroscopic release. Two cases of post-operative minimal hematomas, 2 cases of bruising and 2 cases of local subcutaneous edema were observed as early complications and were cured by conservative treatment. After surgery, all incisions healed in stage I, and no other complications such as wound infection, nerve and blood vessel injury were detected. One hundred eighteen patients were followed up for 6 to 72 months (mean, 36 months). No lateral instability of the hip was observed and all patients returned to normal gait. The degree of adduction of the hip joint in all these 118 patients was significantly improved relative to their pre-operative conditions. One hundred fifteen patients (97.5%) were able to crouch with knees close to each other after surgery. One hundred fourteen patients (96.6%) were able to cross the affected leg completely without any support. The GD scale was improved from 55.5 ± 10.6 before operation to 90.1 ± 5.2 at the last follow-up (*p* < 0.05). The patient satisfaction rate was 95.8%.

**Conclusion:**

Arthroscopic tight fibrous band release using anterior and posterior portals is minimally invasive for adult moderate-to-severe gluteal fibrosis, with a high success rate, quick recovery after surgery and reliable medium-term effect.

## Background

Acquired gluteal fibrosis (GF) is a clinical condition characterized by contracture of the gluteal muscles, tensor fascia lata (TFL), iliotibial band (ITB), and the related fascia; in severe cases, it can also involve hip external rotators and, rarely, the hip joint capsule [1, 2]. This condition was first described by Fernandez de Valderrama in 1969 [3]. In China, a large number of GF cases reported around the year 2000 were children and were usually associated with repeated intramuscular injection into the gluteal region [3, 4]. Epidemiological investigations in China show that the incidence of GF ranges from 0.7 to 10.1% [5–8]. Alongside the prohibition of benzyl alcohol as a dissolvent for penicillin for intramuscular injection in 2005 and the decrease of gluteal intramuscular injection, the number of new GF cases has shown a decreasing trend in the recent decade in China. At present, the existing cases are mainly the adult patients left over from the past.

Patients with GF typically present with abducted and externally rotated hip. They are usually unable to bring both knees together when crouching and to cross the affected leg completely without any support [9]. Exercises and stretching of the shortened muscles do not produce notable improvement [3], while arthroscopic release of GF has shown remarkable improvement in symptoms and quality of life for the patients [2, 10–12]. However, the technique and outcomes of arthroscopic release for moderate and severe GF in adults are rarely discussed in the previous reports. For patients with severe contracture, surgeons are generally concerned about the efficacy and safety of the arthroscopic release itself.

The objective of this study was to evaluate the clinical outcomes of arthroscopic tight fibrous band release in the treatment of adult moderate-to-severe gluteal fibrosis using anterior and posterior portals during mid-term follow-up.

## Methods

### Inclusion and exclusion criteria

All adult patients with moderate GF or severe GF were considered for inclusion. The exclusion criteria were: (i) patients who were < 18 years of age with GF; (ii) patients who were > 50 years of age with GF; (iii) patients who belonged to mild GF; (iv) patients with a history of fracture in the affected hip; (v) the affected side of the hip suffering from moderate to severe arthritis, hip dysplasia or femoral head necrosis.

### Patients’ information

**Fig. 1:**
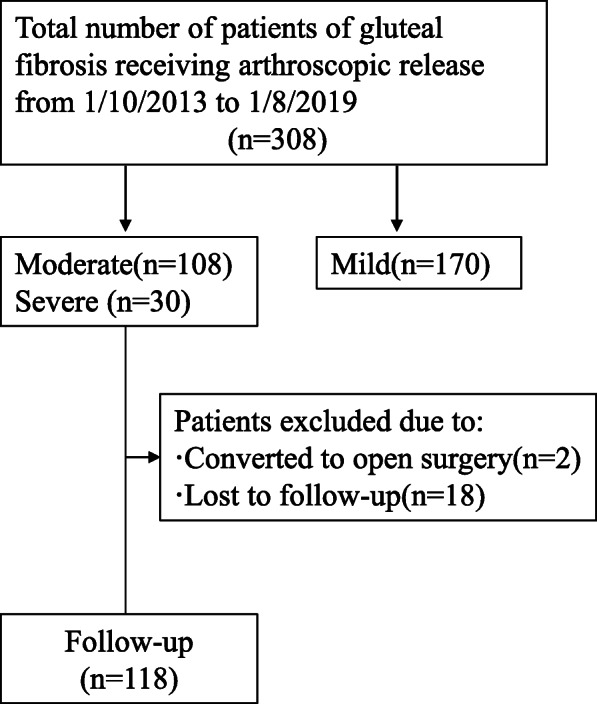
Flowchart: 18 patients were lost to follow-up and the number of eligible patients was 118

The cases reviewed in this retrospective study were recorded between October 2013 and August 2019. A total of 308 patients who underwent arthroscopic tight fibrous band release at the authors’ institution were included eventually. We excluded 170 patients with mild GF or younger than 18 years. With the exclusions, we had 138 patients (276 hips; all were bilateral) (Fig. 1). The patients’ characteristics are summarized in Table 1. Specifically, there were 58 men and 80 women with a mean age of 28.6 years (range, 18–42 years). The history of the patient’s hip intramuscular injection is 100% (both benzyl alcohol and penicillin). There are 20% of conservative treatment patients, the treatment method is rehabilitation treatment, the treatment time is 6–12 months and 4 cases are treated with small needle-knife therapy. No cases of collagen-related diseases. The study protocol was approved by the Institutional Review Board of the authors’ institution. Informed consent was obtained from all participants before carrying out any research work.

**Table 1:** Baseline characteristics and results of the included patients

Variables	
No. of patients	138
Age, mean (range)	28.6 (18–42)
Gender (male/female)	58/80 (42%/58%)
History of hip intramuscular injection	100% (both benzyl alcohol and penicillin)
Snapping symptom	138 (100%)
Duration of symptoms, median (range)	25 years (12 month–35 years)
Operation time, mins, mean (range)	18 (10–30)
Blood loss, ml, mean (range)	4 (2–10)
Total days of hospital stay, days, mean (range)	3.85 (1–7)
Length of postoperative hospital stay, days, mean (range)	1.28 (1–6)
Follow-up No. of patients	118
Follow-up period, months, mean (range)	36 (6–72)
Complications	
Post-operative minimal hematomas, n	2
Bruising, n	2
local subcutaneous edema, n	2
Pre-op adduction, degree, mean ± standard	−46.4 ± 15.6
Post-op adduction, degree, mean ± standard	22.7 ± 5.1*
No. of patients who were able to crouch with the knees close to each other after surgery	115 (97.5%)
No. of patients who were able to cross the affected leg completely after surgery	114 (96.6%)
Pre-op GD scale, mean ± standard	55.5 ± 10.6
Post-op GD scale, mean ± standard	90.1 ± 5.2*
Patient satisfaction rate	113 (95.8%)

* *p* < 0.05, Post-op vs Pre-op

### Characteristics of the GF degree

(i) Mild GF [13]: The extorsion of lower limb is mild; the abduction contracture is less than 15° with both hips and knee joints in 90° of flexion or the adduction range is less than 20° with no flexion. The Ober’s sign and frog squatting sign are weakly positive. The limp gait is not apparent with the lateral inclination of pelvis on the anteroposterior radiograph being less than 10°.

(ii) Moderate GF [13]: The extorsion of lower limb is moderate; the abduction contracture ranges from 15 to 60° with both hips and knee joints in 90° of flexion or the adduction range is less than 10° with no flexion. The Ober’s sign and frog squatting sign are positive. The limp gait is apparent with the lateral inclination of pelvis on the anteroposterior radiograph being less than 20°.

(iii) Severe GF [13]: The extorsion of lower limb is severe; the abduction contracture is more than 60° with both hips and knee joints in 90° of flexion or the adduction range is less than 0° with no flexion. The Ober’s sign and frog squatting sign are strongly positive. The limp gait is remarkably apparent with the lateral inclination of pelvis on the anteroposterior radiograph being more than 20°.

Among all the included patients, 108 belonged to moderate GF and 30 belonged to severe GF according to the classification standard of gluteal fibrosis [10].

### Surgical technique

#### Anesthesia and preoperative skin mark

**Fig. 2:**
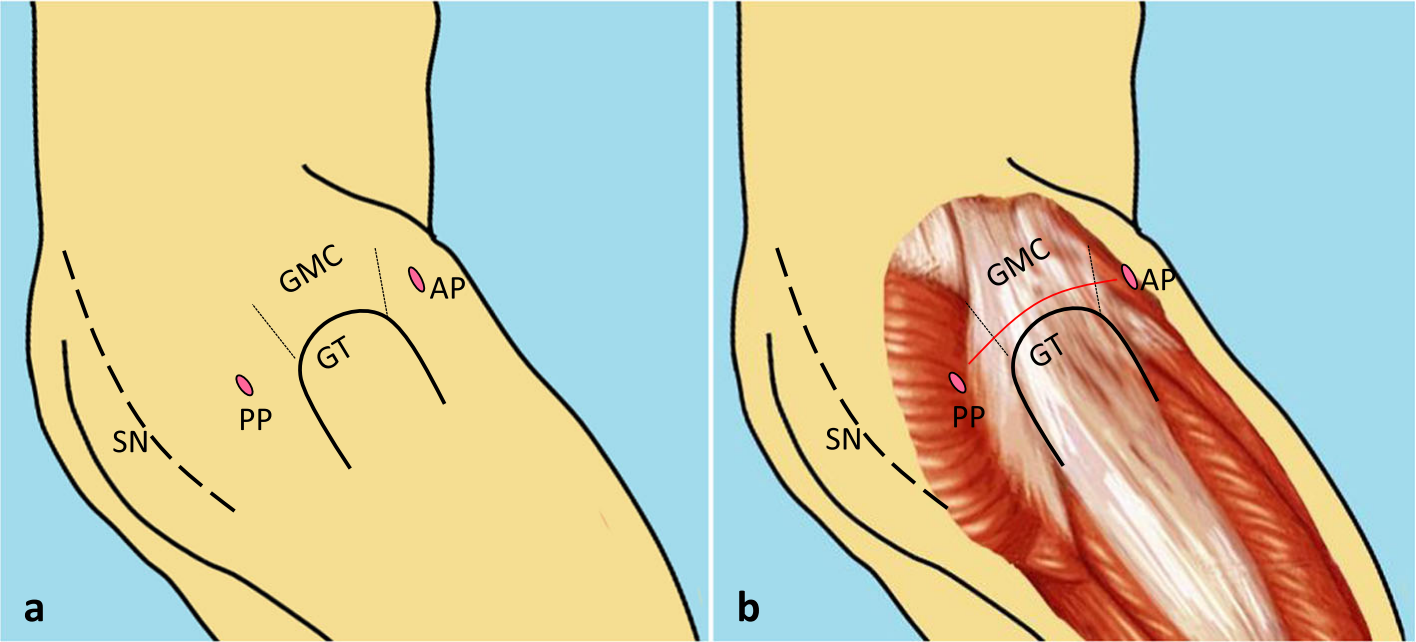
**a**: The diagram shows the important anatomic landmarks on the patient during surgery. AP = anterior portal; PP = posterior portal; GMC = gluteal muscle contracture; GT = great trochanter; SN = sciatic nerve. **b**:Diagrammatic representation (red line) of arthroscopic release of GMC above the GT. This picture was drawn by the corresponding author

General anesthesia or spinal anesthesia was executed in the operation process with the patient being placed in a lateral position. A physical examination was conducted preliminarily under different flexion angles and adduction degrees of hip joint before operation to determine the extent and severity of contracture. The anterior and posterior borders of the gluteal muscle contracture (GMC), the greater trochanter (GT) and the sciatic nerve (SN) were marked before operation (Fig. 2a, b). A two-portal technique, namely the anterior portal (AP) and posterior portal (PP), was used to perform the operation (Fig. 2). These two portals (AP and PP) were located in front of and behind the top of the greater trochanter respectively.

Alternatively, the AP could be placed in front of the anterior edge of the GMC and the PP could be placed behind the posterior edge of the GMC. The position of PP was adjusted based on the position of the contractile bands determined before surgery and the expected release area. For instance, if the contractile bands were relatively posterior, the PP would be adjusted posteriorly along the horizontal line of the greater trochanter. The sciatic nerve was at least 2 cm distal to the PP. The distance between the two approaches was about 10 cm. The line connecting the two portals was roughly perpendicular to the femoral shaft.

#### Approach and exposure

A 30 ml mixture of 0.01% adrenaline, normal saline and ropivacaine was injected between the GMC and the subcutaneous tissue to reduce bleeding, keep the arthroscopic view clear and relieve postoperative pain. The operative portals (AP and PP) were established according to the preoperative mark. The surface of GMC was separated from the subcutaneous fat by a periosteal dissector to form a 2 cm × 10 cm cavity, which provided a good working space.

During the operation, 3000 ml of normal saline supplemented with 1 ml of 0.1% adrenaline was used for continuous gravity perfusion, which was beneficial to hemostasis and maintenance of a clear operative field. The PP was used as the viewing portal, while the AP was used as the working portal for instruments such as shaver and radiofrequency device. The subcutaneous adipose tissue overlying the fibrous band of the gluteal muscle group that obstructed the operative view was removed by a shaver through the AP, so that the anterior and posterior borders of the gluteal muscle group could be clearly observed.

#### GMC release

The GMC, which was confirmed by arthroscopy, was different from the normal muscle tissue. Its structure was usually similar to the white scar tissue. The tight fibrous band around the trochanter major was sectioned from anterior to posterior port by a radiofrequency device through the AP (Fig. 2b). The radiofrequency device was also used to coagulate any bleeding point to observe the operation site clearly during the entire procedure and to prevent the formation of post-operative hematoma.

The Ober’s test was carried out and the sliding of the contraction band was observed under arthroscopy to determine the degree of release and the position of the residual contraction band. If passive adduction was limited in flexion, it was usually necessary to release the GMC behind the great trochanter. If the adduction was limited in the extended position, it was usually necessary to release the GMC in front of the greater trochanter. The degree and depth of GMC release were adjusted according to the Ober’s test and arthroscopic observation.

If passive adduction was still limited after the release of the fascia lata and gluteus maximus, the contractile bands of gluteus medius would be explored and then released selectively. Damage of the attachment of gluteus medius in the greater trochanter should be avoided as far as possible to maintain the hip abductor muscle strength and the hip joint function after operation. If the contraction band was relatively posterior, making the operation difficult, the hip joint should be flexed to slide the contraction band anteriorly. The sciatic nerve should always be cautiously taken care of during the operation and efforts should be made to ensure that the release was not too posterior or deep.

The contracture of gluteus minimus and hip joint capsule was found and released in a few serious cases. Then, any residual deformities were evaluated carefully. The complete release of contracture was confirmed by flexion, adduction, internal rotation, palpable click, the Ober’s sign and the cross leg sign.

#### Postoperative management

Postoperative hemostasis was achieved by using a lateral position or ice bag compression. The patients were allowed to flex their hips and knees and cross their legs at 30-min intervals as tolerated on postoperative day 1. The legs had to be crossed as much as possible to extend the released GMC and to minimize the possibility of hematoma formation after surgery. A rehabilitation program which included gradual full range of hip movement and stretching of GMC (e.g., walking in a straight line, crouching with the knees close together and sitting with stacked legs) was initiated on postoperative day 2 and would continue for 3 months. These exercises were performed three to five times a day with 10 to 30 repetitions depending on the patients’ endurance.

#### Postoperative evaluation

Postoperative hematoma, ecchymosis under the skin and early wound complications were observed. The operation time, length of hospital stay and surgery-related complications, such as neurovascular injury and wound complications, were recorded. The gluteal muscle contracture disability (GD) scale (Table 2) [14] and the subjective satisfaction of patients were used to assess the outcomes at the last follow-up.

**Table 2:** The gluteal muscle contracture disability (GD) scale [2]*

Item	Score	Item	Score
1. Walk with a toe-out gait		8. Hip fatigue	
Very serious, affecting walking	1	·Obvious during daily activities	1
Obvious, affecting sporting exercises	4	·Yes, during regular exercises	2
Only a slight gait change	7	·Yes, during intensive exercises or long-time squatting	3
No gait change	10	·No hip fatigue	4
2. Ability to cross or overlap the legs		9. Hip friction or pain	
Unable	1	·Very serious during daily activities	1
The ankle can just reach the opposite knee	4	·Yes, during regular exercises	2
The lower leg can reach the opposite knee	7	·Yes, during intensive exercises or long-time squatting	3
Normal	10	·No hip friction or pain	4
3. Ability to crouch with the knees close to each other		10. Upright sitting posture	
Unable	1	·The upper body cannot stay upright	0
Restricted, able to crouch on tiptoes	4	·The upper body can stay upright, but the knees cannot bring together	2
Able to crouch in great effort with both feet fully landing on the floor and close to each other	7	·The upper body can stay upright with the knees bringing together	6
Unrestricted	10	11. Whether or not the knees can bring together when the patient is in a lateral position with the legs extended?	
4. Snapping hip		·No	0
Yes, during walking	1	·Yes	10
Yes, during Squatting	4	12. Whether or not the ankles can cross over when the patient is in a supine position with the legs extended and crossed over?	
Yes, during hip flexion, internal rotation and adduction	7	·Yes	0
No	10	·No	10
5. Buttock shape		13. Whether or not running is affected?	
Sharp buttocks	1	·Yes	0
Flat buttocks	4	·No	2
Slight atrophy of buttocks	6	14. Whether or not standing long jump can be completed?	
Normal	8	·Complete with difficulties	0
6. Whether or not the buttock skin has dimples, depressions and deep grooves?		·Yes	2
Obvious deep groove was observed at any position of the buttock	1	15. Whether or not the hurdling action is affected?	
Depression was observed at hip flexion and adduction	4	·Severely affected	1
Yes, during crouching with the knees close to each other	6	·Moderately affected	2
No	8	·Mildly affected	3
7. Any restriction while climbing up or down stairs?		·Not affected	4
Yes	0		
No	2		

### Statistical analysis

Statistical analyses were performed using SPSS software version 22.0 (SPSS Inc., Chicago, IL, USA). The paired t test was conducted to test for the differences in scores between the preoperative and postoperative measurements. A *p* value < 0.05 was considered as statistically significant.

## Results

### General results

A total of 138 patients with bilateral moderate-to-severe gluteal fibrosis were treated in two centers from October 2013 to August 2019 (Fig. 1). There were 2 severe GF patients began as an arthroscopic procedure, but were converted to open surgery because the arthroscopic release was unsatisfactory. Eighteen patients were lost to follow up. One hundred eighteen patients were followed up for a minimum of 6 months (mean, 36 months; range, 6–72 months).

The duration of procedure for one side ranged from 10 to 30 min; the mean operation time was 18 min (Table 1). The average blood loss was 4 ml (range, 2–10 ml) for unilateral arthroscopic release. The average total length of hospital stay was 3.85 days (range, 1–7 d). The average length of post-operative hospital stay for the arthroscopic surgery group was 1.28 days (range, 1–6 d).

### Complication

Two cases of post-operative minimal hematomas, 2 cases of bruising and 2 cases of local subcutaneous edema were observed as early complications and were cured by conservative treatment (Table 1). After surgery, all incisions healed in stage I. Neither wound infection nor sciatic nerve injury were observed. The strength of the hip abductor muscle was not weakened.

### Functional scores and patient satisfaction rate

**Fig. 3:**
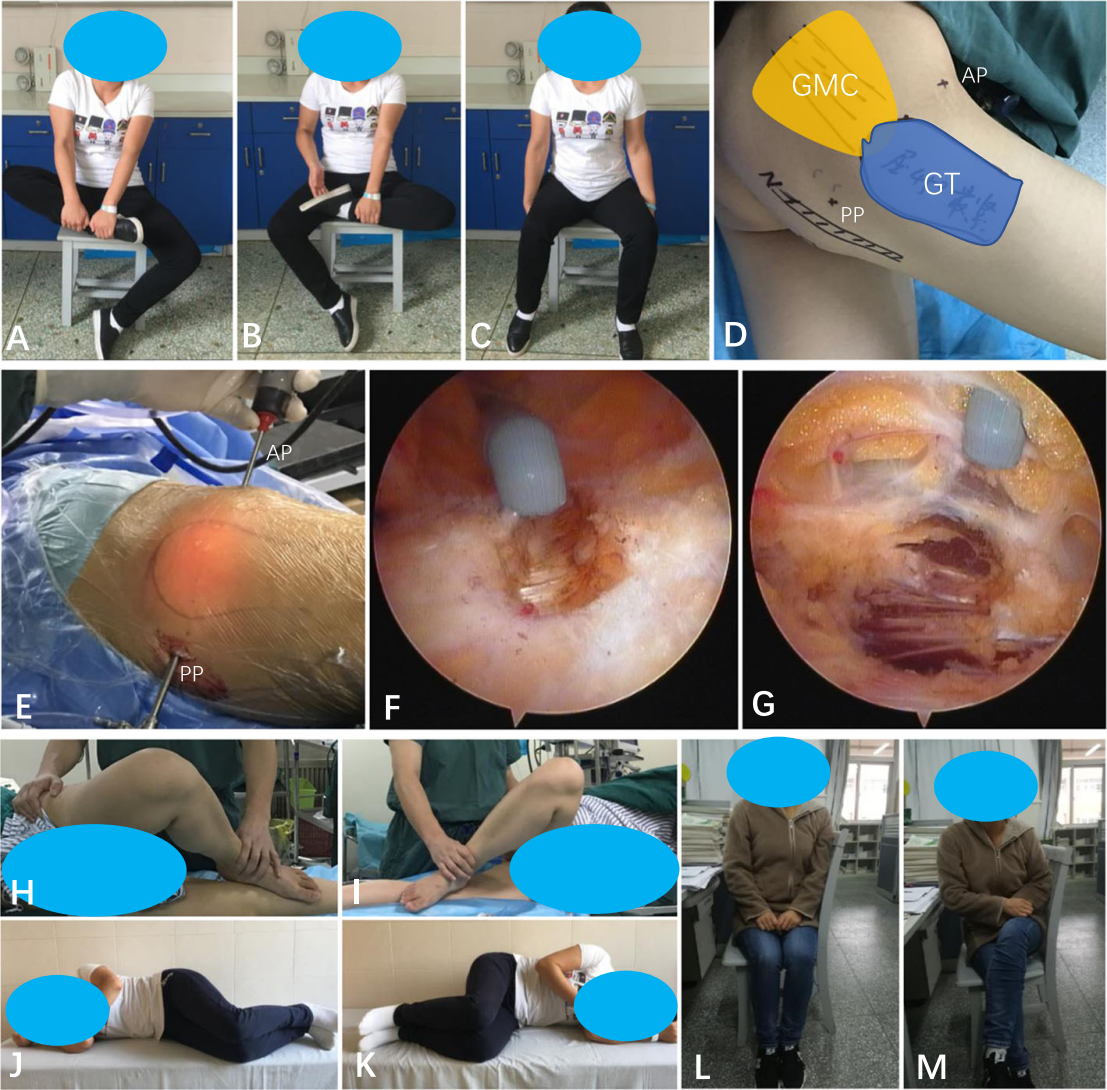
**a**, **b**: A female patient of gluteal fibrosis treated with arthroscopic tight fibrous band release. Before surgery, the patient was unable to cross or overlap his leg while sitting. **c**: The knees could not be kept together while sitting. **d**: Intraoperative photograph showing the important anatomic landmarks on the patient during surgery and the portals for arthroscopic gluteal muscle contracture (GMC) release. **e**: PP indicates posterior portal (the viewing portal), AP indicates anterior portal (the working portal). **f**: The picture suggests the intraoperative view under arthroscopy showing radiofrequency device cutting the tight fibrous band of gluteal fibrosis though anterior portal. **g**: Normal muscle fibers can be seen after tight fibrous band release. **h**, **i**: Ober’s sign was positive. **j**, **k**: Two days post-operative pictures, Ober’s sign was negative. **l**, **m**: At 2-month follow-up, the knees could be kept together while sitting (**l**) and the patient could sit with her legs crossed (**m**)

No lateral instability of the hip was reported. All patients returned to normal gait. The degree of adduction of the hip joint in all the 118 patients was significantly improved relative to their pre-operative conditions (*p* < 0.05) (Table 1). One hundred fifteen patients (97.5%) were able to crouch with the knees close to each other after surgery. One hundred fourteen patients (96.6%) were able to cross the affected leg completely without any support. The GD scale was improved from 55.5 ± 10.6 before operation to 90.1 ± 5.2 at the last follow-up (*p* < 0.05). The patient satisfaction rate was 95.8%. For more details of the whole procedures in our study, please refer to a typical case (Fig. 3).

## Discussions

### Characteristics of adult gluteal fibrosis

GF appeared to be caused by multiple factors. Intramuscular injection of antibiotics, vitamins, antipyretic, benzyl alcohol, quinine or analgesics is the common etiology that leads to gluteal muscle contracture, which can cause direct muscle damage and subsequently the development of muscle fibrosis in the affected area [15, 16]. Except for the injection history, GF is also related to other factors such as scar constitution, poliomyelitis, history of abscesses in buttocks, idiopathic GMC, cytokines (e.g., TGFβ-1, β-3, Smad4, and sphingosine-1-phosphate), immune function abnormalities, physical relationship, as well as children’s susceptibility factors, character factors, gender factors and genetic factors [1, 16–18]. 100% of our patients involved in this study had an injection history of penicillin dissolved with benzyl alcohol. However, the specific frequency and dosage of injection were unknown. This is probably because most of our patients received the injections in the 1980s and 1990s when they were teenagers and were not able to recall the history.

Children and teenagers were the most common patients of gluteal fibrosis or gluteal muscle contracture in the previous literature. Liu et al. [19] reported the use of radiofrequency vaporization under arthroscopy to treat injection gluteus contracture for 18 patients in 2003. The average age of their patients was 14 years old. Yuan et al. [20] compared the clinical efficiency of the treatment of gluteal muscle contracture with arthroscopic release and conventional open surgery based on 18 patients in 2006. The average age of their patients was 9.45 years old. The initial symptoms of gluteal muscle contracture mainly include changes in gait, such as splay foot walking and body shaking during running. Most of the patients cannot cross their legs actively, and/or have snapping hip. With the growth of age and the progress of disease, most of the patients with gluteal fibrosis would experience a certain degree of bone development abnormalities, such as pelvis tilt, pelvis dense belt, change of iliac bone walking direction, narrowing and lengthening of pelvis, and increase of femoral neck stem angle. At present, most of the patients with GMC were adults. In our clinical observation, we found that a part of adult patients also exhibited some other changes, such as pseudo leg length discrepancy and spinal compensatory scoliosis, resulting in abnormal gait. It was common for adult patients to worry about the effect of surgical treatment before operation. In our study, all patients were adults, with a minimum age of 18 years old and a maximum age of 42 years old (mean, 28.6 years). The follow-up results showed that all the patients achieved good results. The functions of internal rotation, adduction and internal rotation of hip joint were basically restored to normal after operation in 3 patients over 40 years old. Therefore, we supposed that arthroscopic release of gluteal fibrosis in adult patients could also achieve satisfactory results.

There are two main reasons why the diagnosis and treatment were made at such a late age. First of all, these symptoms did not affect the patients’ basic living condition when they were young. They could walk normally, and most of the patients could also run and even engage in vigorous sports such as basketball and football. Thus, neither the patients themselves nor their family realized that this might be a disease and they might need medical treatment. Second, most of these patients resided in remote villages in China when they were young, where the medical and healthcare conditions were relatively backward. Some of the patients did not receive correct diagnosis and proper treatment after seeing a doctor. Some of the patients were though diagnosed with gluteal muscle contracture long ago, but open surgery was generally executed in the past. Some other patients had concerns over the complications of operation and the large wounds left on the buttocks, and therefore were not willing to undergo surgery. Nowadays, it is possible to perform minimally invasive release surgery under arthroscopy, which has less trauma, quick recovery, small wounds, and fewer complications. Many of the patients who were afraid of open surgery before are now willing to accept minimally invasive surgery.

### Classification of gluteal fibrosis

The location, range and depth of gluteal muscle contracture varied in different patients. In our clinical work, we found that the severity of contracture obviously affected the effect of arthroscopic release of gluteal fibrosis. Many scholars have studied and discussed the classification and graduation of gluteus contracture, but no widely accepted and applied typing method in clinical practice has been agreed yet. In 2009, Zhao et al. [13] summarized 172 cases of gluteal muscle contracture undergoing open surgery. The patients were classified into three levels (Level I = mild, Level II = moderate, Level III = severe) (typical type and special type) and three types, including Type MA (Gluteus maximus contraction type), Type MEI (Gluteus medius and minimus contraction type) and Type AGM (Gluteus maximus, medius and minimus contraction type). In 2012, Ye et al. [21] introduced a new minimally invasive method for surgical release in 1059 consecutive patients with gluteal muscle contracture. Their patients were assigned to 4 categories: type A, contracture occurred mainly in the iliotibial tract; type B, contracture occurred in the Iliotibial tract and gluteus maximus; type C1, movement of the contraction band was palpable accompanied by an audible snapping sound during squatting; and type C2, movement of the contraction band was not palpable or almost absent accompanied by an audible snapping sound during squatting. In 2013, Liu et al. [22] reported the treatment for a total of 358 patients with gluteus contracture. According to the clinical characteristics and intraoperative situation, their patients were classified into four groups: cable strip, fan-shaped, mixed, and tensor fasciae latae contracture. The aforementioned classification systems did not seem to differ significantly from each other and all these classification systems were practically reliable in understanding the disease pathology and useful in choosing the correct treatment options. The classification system proposed by Liu et al. [13] was based on all types of contractures at different levels with a focus on the functional and pathologic changes, and thus it was adopted to assess the degree of gluteal fibrosis in our study. We found that most of the moderate-severe GMC patients were tensor fascia lata combined with gluteal maximus muscle contracture or mixed type. For such cases, the extent and depth of the release were relatively high, and the difficulty of operation increased significantly. In the previous reports of arthroscopic GMC release, most of the contracture cases were not separated from moderate-severe contracture cases. Therefore, the effect of arthroscopic treatment in moderate-severe GMC patients was not clear. In this study, 118 patients with moderate-severe GMC were followed up and the results showed that arthroscopic tight fibrous band release also achieved good results for adult moderate-to-severe gluteal fibrosis.

### Treatment of gluteal fibrosis

Non-operative treatments, including massage, physiotherapy, shortwave diathermy, and active and passive stretching exercises, were only suitable for mild cases, or recommended for patients who were not eligible for surgery or were waiting for surgery [23]. Once the contracture was established, non-surgical treatments would be useless [21, 23]. Surgical treatment was the gold standard for all the established GMC cases. Some scholars claimed that open surgery was generally suitable for releasing various degrees and types of GMC, particularly for severe (grade III) GMC, while arthroscopic surgery was mainly suitable for mild GMC (grade II) with a relatively limited area [23–29]. In the meantime, some other scholars thought that the new minimally invasive open release could be considered in all cases of GMC [21]. When performing this operation, the surgeon must have comprehensive knowledge and skills of the anatomical signs and procedures, because the complete segmentation of the contracture belt is the main part of the operation [21]. Although this operation seems simple and easy to perform, the surgeon should keep in mind that it is a blind procedure and has full chances of complications [23].

The success of surgical treatment lies in two points: complete release of fibrous tissue and protection of normal tissue. Incomplete release will lead to residual symptoms and dissatisfaction, while excessive release will damage the stability of the hip joint, which may lead to Trendelenburg gait. Arthroscopic release was not recommended as the primary treatment for level III patients in previous studies. The reason might be related to the operative approach itself. In most literature, the two portals of arthroscopy were longitudinally arranged on the outside of the trochanter and buttock and were located at the proximal and distal side of the buttock. The line connecting the two portals was roughly parallel to the femoral shaft. A large area of subcutaneous tissue was dissociated in operation so as to provide enough operation space. An artificial working space (6 cm × 8 cm) was created in the interval between the subcutaneous fascia and the contracture bands [2]. For level III patients with severe contracture, the artificial working space needs to be larger enough to obtain better exposure of the contracture band. The wide range of subcutaneous separation during the operation can easily cause wound bleeding, which will affect the operative field and increase the operation time and the incidence of complications, such as postoperative wound hydrops and hematocele.

Our arthroscopic portals were different from those reported in previous literature. The two portals (AP and PP) were located in front of and behind the top of the greater trochanter respectively, and the position of PP was adjusted based on the position of the contractile bands determined before surgery and the expected release area. For instance, if the contractile bands were relatively posterior, the PP would be adjusted posteriorly along the horizontal line of the trochanter major. The sciatic nerve was at least 2 cm distal to the PP. The distance between the two approaches is about 10 cm. The line connecting the two portals was roughly perpendicular to the femoral shaft. An artificial working space (2 cm × 10 cm) was created. The tight fibrous band (fascia lata, iliotibial tract and gluteus maximus) around the trochanter major was sectioned from anterior to posterior port by a radiofrequency device through the AP. Then, any residual deformities were evaluated carefully. The complete release of contracture was confirmed by flexion, adduction, internal rotation, palpable click, the Ober’s sign and the cross leg sign. In most cases, releasing tight fibrous band of the fascia lata and gluteus maximus were sufficient. If passive adduction was still limited after the release of the fascia lata and gluteus maximus, the contracture bands of gluteus medius would be explored and then released selectively. If passive adduction was still limited after the release of gluteus medius, the contracture bands of gluteus minimus and hip joint capsule would be explored and then released selectively. If passive adduction was still limited after the release of gluteus minimus and hip joint capsule, open treatment was needed. In case of uncontrolled bleeding or deep contracture that was not reachable with an arthroscopic instrument, a small incision should be used. In our study, 2 cases of type III gluteal fibrosis were excluded because the arthroscopic release was unsatisfactory and converted to an open surgery. After the selective release of the deep layer of gluteus medius, the contracture band of gluteus minimus and the partial contracture of the hip joint capsule, satisfactory results were obtained. In Zhao et al.’s study, operative treatment was performed in I to III level patients. All patients in the operative treatment group achieved excellent or fair results, but a number of complications were found in this group (only in level II and III patients), including scar swelling, hematomas, infections, and wound dehiscence [13]. In Amrit S′ study, the patients were classified into type I to IV according to the classification for the location of contraction of external snapping hip, and the excellence rate of surgery reached 100% in type I and type II and reached 92.7% in type III. No long-term postoperative complications were found and no infections, major swelling, hematomas, or wound dehiscence occurred in all of the four types of cases. All patients achieved good results after arthroscopic surgery [30]. For the 118 patients in our study, the GD scale was improved from 55.5 ± 10.6 before operation to 90.1 ± 5.2 at the last follow-up (*p* < 0.05), and the satisfaction rate was up to 95.8%. Comparing our results with Zhao et al.’s and Amrit Set al.’s results, it can be found that patients with GMC can obtain positive outcome after both open and arthroscopic surgery, and can achieve not only complete contracture release but also a lower incidence of complications alongside a higher cosmetic satisfaction rate under the arthroscopic treatment.

### Limitations

Several limitations of the present study deserve comments. Firstly, our analysis was limited by non-comparative reports of surgical results. Secondly, our study was subject to a particular selection bias as the decisions on surgical option were at the discretion of the chief operating surgeons. The results presented in this study were collected from two hospitals, which may also generate regional and institutional bias. Despite these limitations, our series of cases was fairly large with 118 patients (236 hips, bilateral) and the follow-up was at least 6 months. Nevertheless, further prospective studies should be done to confirm our results. Finally, our study was in lack of objective evaluation of changes in the gait pattern and hip abductor strength.

## Conclusion

In conclusion, arthroscopic tight fibrous band release using anterior and posterior portals is minimally invasive for adult moderate-to-severe gluteal fibrosis, with a high success rate, quick recovery after surgery and reliable medium effect. The key to the success of the treatment of adult moderate-to-severe gluteal fibrosis includes complete release of tight fibrous band, complete hemostasis during operation and active rehabilitation after surgery.

## Data Availability

The datasets used and analyzed during the current study are available from the corresponding authors on reasonable request.

## Abbreviations

GF: Gluteal fibrosis
GD: Gluteal muscle contracture disability
TFL: Tensor fascia lata
ITB: Iliotibial band
GMC: Gluteal muscle contracture
GT: Greater trochanter
SN: Sciatic nerve
AP: Anterior portal
PP: Posterior portal

## References

[1] Alves K, Katz JN, Sabatini CS (2019). Gluteal fibrosis and its surgical treatment. J Bone Joint Surg Am.

[2] Rai S, Jin S, Meng C, Chaudhary N, Tamang N, Wang X, et al. Arthroscopic release using F and C method versus conventional open release method in the treatment of gluteal muscle contracture: a comparative study. BMC Musculoskelet Disord. 2017. 10.1186/s12891-017-1484-6. 10.1186/s12891-017-1484-6 PMC5356281 28302115

[3] Fernandez de Valderrama JA, Esteve de Miguel R (1981). Fibrosis of the gluteus maximus: a cause of limited flexion and adduction of the hip in children. Clin Orthop Relat Res.

[4] Sirinelli D, Oudjhane K, Khouri N (1990). Case report 605: gluteal amyotrophy: a late sequela of intramuscular injection. Skelet Radiol.

[5] He X, Li H, Wang D (2003). Classification and management of the gluteal muscles contracture. Chin J Orthop.

[6] Wang B, He X, Wu Y, Fang D, Liu J (2003). The prospective study and factors analysis for gluteus contracture. China J Orthop Traumatol.

[7] Wang Y, Shi J, Chen W, Gao Y (2015). The epidemiological investigation of gluteal muscle contracture of male students in the physical examination for navy pilot recruitment. Chin J Aerospace Med.

[8] Hu X, Tan X, Zheng M, Kuang R, Liang J, Wei W (2015). Epidemiological survey of gluteal muscle contracture of primary and secondary students in Rongchang county. Chongqi Med.

[9] Chen CK, Yeh L, Chang WN, Pan HB, Yang CF (2006). MRI diagnosis of contracture of the gluteus maximus muscle. Am J Roentgenol.

[10] Liu YJ, Wang Y, Xue J, Lui PP, Chan KM (2009). Arthroscopic gluteal muscle contracture release with radiofrequency energy. Clin Orthop Relat Res.

[11] Zhang X, Jiang X, He F, Liang Z, You T, Jin D (2017). Arthroscopic revision release of gluteal muscle contracture after failed primary open surgery. Int Orthop.

[12] Dai Z, Chen Z, Liao Y, Tang Z, Cui J (2018). Comparison of arthroscopic versus open surgery on external snapping hip caused by gluteal muscle contracture. Hip Int.

[13] Zhao CG, He XJ, Lu B, Li HP, Wang D, Zhu ZZ (2009). Classification of gluteal muscle contracture in children and outcome of different treatments. BMC Musculoskelet Disord.

[14] Tang XY, Liu YJ, Li CB, Qi W, Qu F, Li HF (2017). Reliability and validity of gluteal muscle contracture disability scale. Orthop J China.

[15] Cai JH, Gan LF, Zheng HL (2005). Iliac hyperdense line: a new radiographic sign of gluteal muscle contracture. Pediatr Radiol.

[16] Sinha S, Gupta S, Kanojia RK (2019). Bilateral gluteus Maximus contracture in a young child: a case report and review of literature. J Orthop Case Rep.

[17] Lu HH, Liu GH, Yang SH (2007). Research development of etiology and diagnosis of gluteal muscle contracture. Orthop J China.

[18] You T, Zang XT, Zhang WT, Jiang XC, Zhang HL, Zuo JW, et al. Research progress on the pathogenesis of gluteal muscle contracture. 2016;24(13):1201–3 (In Chinese).

[19] Liu YJ, Wang ZG, Li ZL, Zhang WT, Wang Y, Chen JY (2003). The release of gluteal muscle contracture by radiofrequency vaporization under arthroscopic guidance. Chin J Orthop.

[20] Yuan P, Wang WC, Chen Y, Zhang ZF (2006). Comparison of the therapeutic effects of arthroscopic treatment and conventional therapy for gluteal muscle contracture. Orthop J China.

[21] Ye B, Zhou P, Xia Y, Chen Y, Yu J, Xu S (2012). New minimally invasive option for the treatment of gluteal muscle contracture. Orthopedics..

[22] Liu YJ, Wang ZG, Wang JL, Li SY, Li HF, Qu F (2013). Clinical classification of gluteal muscle contracture under arthroscopy. Zhongguo Gu Shang.

[23] Rai S, Meng C, Wang X, Chaudhary N, Jin S, Yang S (2017). Gluteal muscle contracture: diagnosis and management options. SICOT J.

[24] Zhang T, Xu S, Li H, He X, Zhang F (2018). Comparison of the clinical effects of arthroscopic surgery vs. open surgery for grade II glutealmuscle contracture in adults. Exp Ther Med.

[25] Ekure J (2007). Gluteal fibrosis. A report of 28 cases from Kumi hospital, Uganda. East Cent Afr J Surg.

[26] Cui JC, Wang WC, Wu B, Wang SY (2008). Release of gluteal muscle contracture by radiofrequency under arthroscopy. Zhong Nan Da Xue Xue Bao Yi Xue Ban.

[27] Fu D, Yang S, Xiao B, Wang H, Meng C (2011). Comparison of endoscopic surgery and open surgery for gluteal muscle contracture. J Pediatr Orthop.

[28] Xu J, Geng X, Muhammad H, Wang X, Huang JZ, Zhang C (2014). Comparison of the incisions for the open surgical treatment of gluteal muscle contracture. J Pediatr Orthop B.

[29] Chen HS, Yang XL (2015). Insertion of gluteus maximus tendo-chilles lengthening with Z-shaped for the treatment of severe gluteal muscle contracture. Zhongguo Gu Shang..

[30] Shrestha A, Wu P, Ge H, Cheng B (2017). Clinical outcomes of arthroscopic surgery for external snapping hip. J Orthop Surg Res.

